# Advances in the control of vaccine preventable diseases in Ethiopia

**DOI:** 10.11604/pamj.supp.2017.27.2.12701

**Published:** 2017-06-09

**Authors:** Liya Wondwossen, Kathleen Gallagher, Fiona Braka, Thomas Karengera

**Affiliations:** 1 Federal Ministry of Health, Ethiopia; 2 World Health Organization, Ethiopia Country Office; 3 Currently with CDC-Ethiopia; 4 Currently with World Health Organization, Nigeria Country Office

**Keywords:** vaccine preventable diseases, Ethiopia, Expanded Program on Immunization, disease control

## Editorial

Tremendous advances in the past decade have been made in the development and introduction of new vaccines and expansion of immunization programs to reach every child. These advances, in combination with other health care and development interventions such as improved hygiene, sanitation, and education have led to significant reductions in the number of deaths in children under 5 years of age annually. Despite increases in the annual birth cohort globally, these childhood deaths fell from an estimated 9.6million in 2000 to 7.6 million in 2010 [1]. Increased immunization coverage has helped drive this reduction in childhood mortality. However, gaps in immunization coverage still persist between and within many countries.

To address these gaps, the Global Vaccine Action Plan (GVAP) was endorsed by the 194 Member States of the World Health Assembly in May 2012 to provide a framework for the prevention of millions of deaths due to vaccine preventable diseases through 2020 by providing more equitable access to vaccines for people in all communities. The GVAP has four major goals: 1) strengthen routine immunization, 2) accelerate the control of vaccine-preventable diseases (with polio eradication as the first milestone), 3) introduce new and improved vaccines and spur research and development.

In Ethiopia, the Expanded Program on Immunization (EPI) was started in 1998 and currently ten antigens (BCG, diphtheria, pertussis, tetanus, Hib, HepB, polio, measles, rotavirus, PCV) are included in the routine childhood immunization schedule. Since 1996, Ethiopia has been implementing polio eradication initiative activities using standard World Health Organization(WHO) recommended strategies. Ethiopia adopted an accelerated measles control strategy in 2001 and the African Regional Measles elimination strategy in 2012. Since 2014, Ethiopia has been implementing a routine immunization improvement plan to further enhance its ability to reach every child with vaccine. Gradual improvements in immunization coverage have been reported with time. In 2015, the WHO-UNICEF joint estimates for DPT-3 and MCV-1 coverage nationwide were 86% and 78%, respectively [2]. Ethiopia has achieved its MDG 4 target, namely a reduction of Under-5 Mortality Rate (U5MR) from 204 in 1990 to 59 per 1,000 live births. Infant Mortality Rate Also declined significantly from 123 to 36 per 1,000 live births [3]. Immunization program contributed to the reduction of child mortality through expansion of the service and introduction of new life saving vaccines.

Despite these efforts, surveillance data shows that cases of vaccine preventable diseases such as measles and polio continue to occur in the country, especially in areas with a history of weak routine immunization services. During 2015 alone, more than 17,000 cases of measles were reported from throughout the country. Ethiopia initially achieved interruption of indigenous wild polio virus (WPV) in December 2001, just five years after launching the “Kick Polio out of Africa” campaign. However, the country has experienced numerous separate WPV importations from neighboring countries. The most recent polio outbreak, starting in 2013, was associated with the larger Horn of Africa outbreak and resulted in 10 cases in the Somali Region of the country. The last case of wild polio virus in Ethiopia occurred in January 2014 but the country remains at risk for importations due to low routine immunization coverage in some area, difficult to reach population, highly porous borders and large pastoralist populations.

This supplement presents data and reports that highlight some of the recent efforts and activities on the part of the Ethiopian Government, EPI partners and the World Health Organization to further the understanding and control of vaccine preventable diseases in Ethiopia. Included in this supplement are manuscripts that describe the epidemiology of measles in selected areas of the country, the impact of polio eradication efforts on routine immunization activities and community knowledge in the Somali region of Ethiopia, analysis and assessment of AFP and other surveillance data, determinants of immunization service utilization and routine immunization performance, and the utility of an accountability framework for monitoring polio eradication efforts. These reports represent a small fraction of the actual efforts throughout Ethiopia in support of the EPI program. Ongoing efforts, adequate resources and capacity and new innovations and strategies continue to be needed to ensure that every child throughout Ethiopia has access to life-saving vaccines.

## Competing interests

Authors declare no competing interests. The views expressed in the perspective articles are those of the authors alone and do not necessarily represent the views, decisions or policies of the institutions with which they are affiliated and the position of World Health Organization.
